# Effects of ulinastatin administered at different time points on the pathological morphologies of the lung tissues of rats with hyperthermia

**DOI:** 10.3892/etm.2014.1656

**Published:** 2014-04-01

**Authors:** ZAI-SHENG QIN, PEI TIAN, XIA WU, HONG-MEI YU, NA GUO

**Affiliations:** Department of Anesthesiology, Nanfang Hospital, Southern Medical University, Guangzhou, Guangdong 510515, P.R. China

**Keywords:** ulinastatin, systemic hyperthermia, pulmonary histopathology

## Abstract

Hyperthermia not only directly induces cell injury of body tissues, but also causes the body to release large amounts of inflammatory mediators and cells with extensive biological activities to induce a systemic inflammatory response and immune dysfunction. Thus, hyperthermia causes systemic inflammatory response syndrome, aggravating injuries to various organs. This study aimed to observe the effects of ulinastatin (UTI) administered at different time points on the cellular morphologies of the lung tissues of rats with systemic hyperthermia. A total of 40 male Sprague Dawley rats were randomly divided into five groups: The normal control group (C group), the hyperthermia group without medication (H group), the hyperthermia and UTI pre-treatment group (HU group), the group treated with UTI at 1 h after hyperthermia (HU1 group), and the group treated with UTI at 2 h after hyperthermia (HU2 group). The systemic hyperthermia rat model was established in a heating chamber with a biological oxygen supply. For the HU, HU1 and HU2 groups, UTI (5×10^4^ U/kg) was administered at different time points. For the C and H groups, an equivalent volume of normal saline was administered. During heating, the respiratory frequency and rectal temperature were measured and recorded once every 30 min. After 2.5 h of heating, the wet/dry weight (W/D) ratio of the lung tissues of the rats was measured. Additionally, the cellular morphologies of the lung tissues were observed under light and electron microscopes. The respiratory frequencies and lung tissue W/D ratios of the rats in the various hyperthermia groups were significantly higher than those of the rats in the C group (all P<0.05). The respiratory frequencies and lung tissue W/D values of the HU and HU1 groups were significantly lower than those of the H group (all P<0.05). Under the light microscope, the bronchial surrounding tissues of the HU and HU1 groups were loose, and the majority of the pulmonary alveolar structures were normal; the H and HU2 groups presented a number of changes, including pulmonary interstitial hyperemia, alveolar epithelial swelling and emphysema. Under the electron microscope, it was observed in the type II epithelial cells of the pulmonary alveoli of the H group that the mitochondria were swollen, the cell ridges were shortened, the microvilli were thin and increased, and the alveolar wall was thickened. Also, an increased number of infiltrating neutrophils were visible. In addition, the type II epithelial cells of the HU2 group also presented these changes to different extents and the changes in the HU and HU1 groups were the mildest. These results indicate that the early application of UTI relieves edema and the extent of cell injury of the lung tissue in rats with systemic hyperthermia.

## Introduction

Hyperthermia may be defined as overheating of the body, including heatstroke caused by an over-high ambient temperature, or as an abnormally high body temperature (1). The potential causes of hyperthermia include infection, certain drugs and medications, and brain trauma (1–3). High temperature may be used for tumor treatment, particularly for cancer treatment (1,2), but controversial issues remain in its clinical use (1). Unrelieved hyperthermia is a cause of mortality, particularly in elderly individuals (1,3). Hyperthermia not only directly induces cell injury of body tissues, but also causes the body to release large amounts of inflammatory mediators and cells with extensive biological activities to induce a systemic inflammatory response and immune dysfunction. Thus, hyperthermia causes systemic inflammatory response syndrome (SIRS), aggravating injuries to various organs and ultimately results in multiple organ dysfunction syndrome (MODS) (3,4). A study reported that hyperthermia-induced elevation of the levels of heat shock protein 70 relieved the extent of the pulmonary fibrosis of rats in response to the induction of acute lung injury by lipopolysaccharide (LPS) administration (5). Therefore, the effects of hyperthermia on the lung and its mechanism of action remain unclear at present. In the clinic, cases of fatal hyperthermia caused by various intraoperative factors are frequently reported (6–9), and a comprehensive treatment measure is the key to successful treatment.

Ulinastatin (UTI) has been used in the process of successfully treating a case of malignant hyperthermia (MH) (10). UTI is a typical Kunitz-type protease inhibitor. A number of studies have suggested that UTI may be able to protect against acute lung injuries caused by endotoxins and mechanical damage (11–13). Additional studies have shown that UTI may be able to protect against acute lung injuries induced by LPS in rats (14–16). However, there are a few studies concerning whether UTI has an intervention effect on hyperthermia-induced lung injury (17–19).

The purpose of the present study was to observe the cellular morphological changes of the lung tissue in rats with hyperthermia and the effects of intervention with UTI administration at different time points on cellular morphology. These observations were conducted to explore the mechanism of action by which UTI treats fatal hyperthermia in the clinic and the importance of the time of application.

## Materials and methods

### Experimental animals and grouping

A total of 40 specific pathogen-free Sprague Dawley male rats with body weights ranging from 180 to 220 g were provided by the Experimental Animal Center of Southern Medical University (Guangzhou, China) and randomly divided into five groups, with eight rats in each group. The groups were as follows: The C group (the rats were maintained at room temperature, without medication), the H group (the rats were placed at high temperature, without medication), the HU group (5×10^4^ U/kg UTI was administered to the rats prior to heating), the HU1 group (5×10^4^ U/kg UTI was administered after 1 h of heating), and the HU2 group (5×10^4^ U/kg UTI was administered after 2 h of heating). UTI was provided by Tianpu Biochemical Pharmaceutical Co. Ltd. (Guangzhou, China). This study was conducted in strict accordance with the recommendations in the Guide for the Care and Use of Laboratory Animals of the National Institutes of Health (ninth edition, 2010). The animal use protocol was reviewed and approved by the Institutional Animal Care and Use Committee of Nanfang Hospital, Southern Medical University.

### Indicators and methods

The rats were anesthetized with 3% pentobarbital (Nembutal; 45 mg/kg; SERVA, 921019, Shanghai, China) by intraperitoneal injection and placed into a heating chamber with a biological oxygen supply (the Artificial Climate Simulation Chamber for Animals, developed by the Tropical Medicine Faculty of Southern Medical University). Also, the previously examined rectal temperature was used as the basic value. Subsequently, the rats in all groups other than the C group were heated in the heating chamber at 35°C and a relative humidity of 60%. For the HU, HU1 and HU2 groups, 5×10^4^ U/kg UTI (dissolved in 5 ml normal saline) was administered at the initiation of heating, and after 1 h and 2 h of heating, respectively. For the other two groups, an equivalent volume of normal saline was administered at the beginning of the experiment. After 2.5 h of heating, all rats were removed from the heating chamber and treated as subsequently described. The time at the start of the experiment was expressed as T0. During the experiment, the respiratory frequency and rectal temperature of the rats were measured and recorded once every 30 min. A total of six recordings at different time points during the experiment were conducted and they were expressed as T0–T150, respectively.

### W/D ratio determination of the lung tissue

After removal of the rats from the heating chamber, the rat thoracic cavity of each group (n=8) was opened to remove the left upper lung tissue before perfusion with paraformaldehyde. Water and blood staining on the lung tissue surface was absorbed with filter paper, and the tissue was weighed in a weighing disk (wet weight), dried in a constant temperature oven at 80°C and weighed again (dry weight). Subsequently, the W/D ratio was calculated.

### Acquisition and observation of specimens for light microscopy

Following the opening of the thoracic cavity, buffer solution containing 4% paraformaldehyde was rapidly perfused via the right ventricle. The left lower lung tissue was removed, soaked and fixed in 4% paraformaldehyde for 24 h, conventionally dehydrated with alcohol, embedded with paraffin wax and sectioned into ultrathin slices. The slices were stained with hematoxylin and eosin and were then observed for pathological changes under a Nikon microscope (Nikon TS100-F, Nikon, Tokyo, Japan) and photographed.

### Acquisition and observation of specimens for electron microscopy

The thoracic cavities of three rats from each group were opened to remove the right upper lung tissue, and each tissue sample was torn into small sections with a size of ~1 mm^3^. The tissue sections were soaked in 2% glutaraldehyde (this step was completed within 1 min), fixed with 2% osmic acid, dehydrated with gradient alcohol, embedded with epoxy resin and sectioned into ultrathin slices. The slices were stained with uranium acetate and lead citrate, and were observed and photographed under a H-7500 Transmission Electron Microscope (Hitachi, Tokyo, Japan).

### Statistical analysis

The data were analyzed using SPSS statistical software, version 13.0 (SPSS, Inc., Chicago, IL, USA), and the experimental data were expressed as the mean ± standard deviation. One-way analysis of variance was used for comparisons of the mean value of various parameter indicators among the groups. P<0.05 was considered to indicate a statistically significant difference.

## Results

### Comparisons of the general data

No significant difference in the body weight, basic rectal temperature and rectal temperatures of the rats at various time points were identified among the groups (all P>0.05; Table I).

### Changes of the pulmonary respiratory frequencies and lung tissue W/D ratios of the rats in the various groups

**Figure 1:**
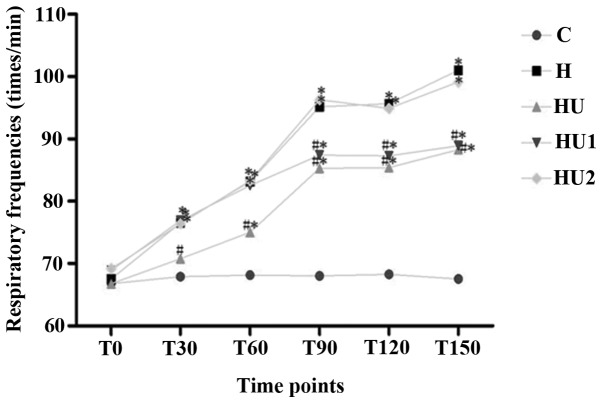
Changes in the respiratory rate of the rats in the different groups. ^*^P<0.05, compared with the C group; ^#^P<0.05, compared with the H group. C, normal control group; H, hyperthermia without medication; HU, hyperthermia and UTI pretreatment; HU1, treated with UTI after 1 h of heating; HU2, treated with UTI after 2 h of heating. UTI, ulinastatin.

**Figure 2:**
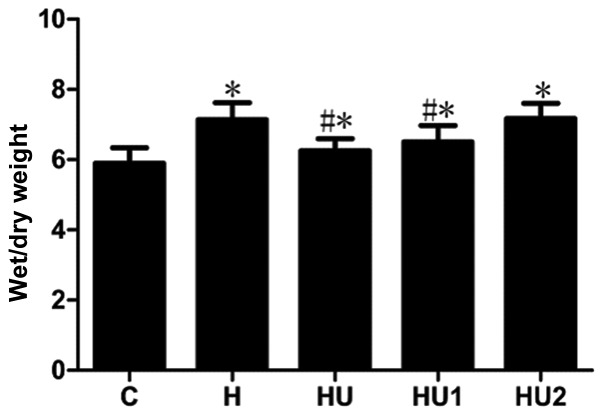
Changes of the W/D ratios of the rats in the different groups. ^*^P<0.05, compared with the C group; ^#^P<0.05, compared with the H group. C, normal control group; H, hyperthermia without medication; HU, hyperthermia and UTI pretreatment; HU1, treated with UTI after 1 h of heating; HU2, treated with UTI after 2 h of heating. UTI, ulinastatin.

During the experiment, the respiratory frequencies and lung tissue W/D ratios of the rats in the various hyperthermia groups were markedly increased. Compared with the those of the C group, the measurements of the hyperthermia groups were significantly different (all P<0.05) with the exception of the respiratory rate of group H1 at 30 and 60 min. In addition, the respiratory frequencies and lung tissue W/D ratios of the HU and HU1 groups were significantly lower than those of the H group (all P<0.05). Between the HU2 and H groups, no significant differences in the respiratory frequency and lung tissue W/D values were identified (P>0.05, Figs. 1 and 2).

### Pulmonary histopathology changes under light microscopy

**Figure 3:**
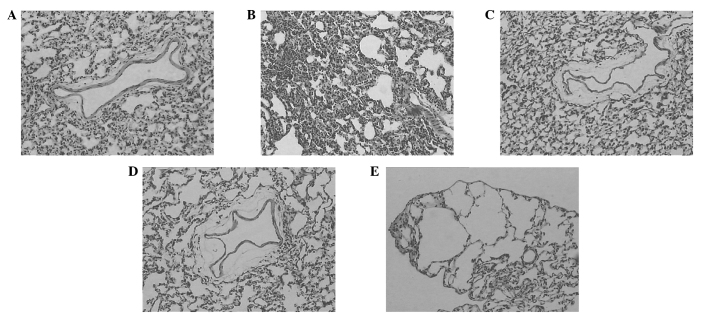
Pathological examination of the lung tissues of the rats in the different groups. (H&E staining, original magnification: ×200). (A) C group; (B) H group; (C) HU group; (D) HU1 group; and (E) HU2 group. C, normal control group; H, hyperthermia without medication; HU, hyperthermia and UTI pretreatment; HU1, treated with UTI after 1 h of heating; HU2, treated with UTI after 2 h of heating. H&E, hematoxylin and eosin; UTI, ulinastatin.

In the C group, the bronchial pulmonary alveoli tissues of the animals were integrated; the bronchial wall did not present hyperemia and edema; the pulmonary alveoli did not present shrinkage or dilation; and no clear exudation in the alveolar space was observed (Fig. 3A). Compared with those of the rats in the C group, the lung tissues of the rats in the hyperthermia treatment groups presented pathological change to various extents. Among them, the pathological changes of the H group (Fig. 3B) were the most severe. The alveolar wall was thickened, twisted and deformed, and pulmonary interstitial hyperemia, and pulmonary alveoli collapsed and patchy atelectasis were observed. Also, pneumorrhagia and emphysema were visible. The pathological changes in the HU and HU1 groups were milder than those of the H group. It was observed that the bronchial surrounding tissues were loose, the alveolar wall presented mild edema, and no clear exudation was visible in the alveolar space (Fig. 3C and D). In the HU2 group, the pulmonary alveolar epithelia were swollen, and the alveolar wall was thinned or broken to form bullae of the lung (Fig. 3E).

### Electron microscopy examination results

**Figure 4:**
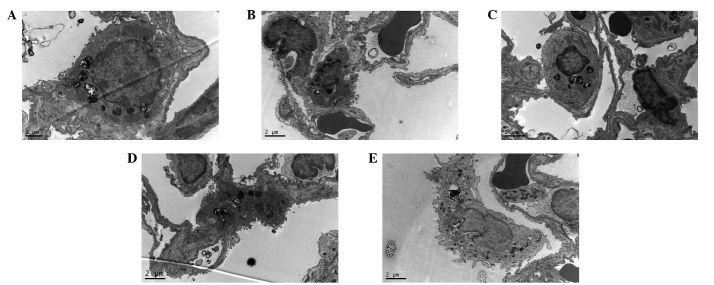
Ultrastructure of the lung tissues of the rats in the different groups. Uranium acetate and lead citrate staining. (A) C group (magnification, ×9,700); (B) H group (magnification, ×5,800); (C) HU group (magnification, ×5,800); (D) HU1 group (magnification, ×5,800); and (E) HU2 group (magnification, ×5,800). C, normal control group; H, hyperthermia without medication; HU, hyperthermia and UTI pretreatment; HU1, administered with UTI 1 h of heating; HU2, administered with UTI 2 h of heating. UTI, ulinastatin.

Under the electron microscope, it was observed in the C group that the pulmonary alveoli were integrated; the nuclear membrane was complete; the nuclear chromatin was uniform; and mitochondria, rough endoplasmic reticula, Golgi apparatus and lysosomes and lamellated bodies arranged in concentric circles or in parallel were present in the cytoplasm of the type II epithelial cells (Fig. 4A). The changes in the H group were the most evident. In the type II epithelial cells of the pulmonary alveoli, the mitochondria were swollen, the cell ridges were shortened, the microvilli were thinned and increased in number, and the alveolar wall was thickened. An increased number of infiltrating neutrophils were visible, and a large number of red blood cell fragments were deposited in the pulmonary alveoli (Fig. 4B). The changes in the HU and HU1 groups were milder than those of the H group. The cell membranes of the type II epithelial cells were complete, and the cytoplasmic organelles were almost normal in structure, which was in line with the observations in the C group (Fig. 4C and D). In the HU2 group, the type II epithelial cells of the pulmonary alveoli had shed and detached from the basement membrane, the lamellated bodies were reduced, and the microvilli were thinned and increased in number (Fig. 4E).

## Discussion

As there are numerous methods of causing an over-high body temperature, there are a number of different damage mechanisms of the body associated with hyperthermia (6–10). Previous studies have shown that high temperature and humidity act as the main factors in the preparation of a hyperthermic animal model of heat stroke (17,18). It was determined from our preliminary experiments that a temperature of 35°C and humidity of 60% satisfied the requirements for model establishment. In this environment, the body temperature of rats rose to 42–43°C in 3 h, and a high survival rate was maintained. The experimental results of the present study showed that compared with those of the C group, the lung tissues of the rats in the various hyperthermia treatment groups presented different extents of pathological changes. Among them, the pathological changes of the H group were the most severe. Under a light microscope, it was observed that the alveolar wall was thickened, twisted and deformed, and pulmonary interstitial hyperemia, and pulmonary alveolar and patchy atelectasis had appeared. Also, pneumorrhagia and emphysema were visible. Under an electron microscope, it was identified that in the type II epithelial cells of the pulmonary alveoli, the mitochondria were swollen, the cell ridges were shortened, the microvilli were thinned and increased in number, and the alveolar wall was thickened. Also, an increased number of infiltrating neutrophils were visible, and a large number of red blood cell fragments were deposited in the pulmonary alveoli. It may be inferred from these results that hyperthermia possibly causes lung tissue damage via the following two mechanisms. One possibility is associated with the direct damage of the cell membrane and intracellular structures caused by hyperthermia. A study (19) reported that a high temperature markedly damaged the close connecting structures of cardiac muscle cells, epithelial cells of the pulmonary alveoli and capillary endothelial cells, and the normal barrier function of the cell membrane was not maintained, as observed by lanthanum nitrate tracer electron microscopy. In the present study, it was observed that hyperthermia caused the type II epithelial cells of the pulmonary alveoli to shed and detach from the basement membrane, the lamellated bodies were reduced, and the microvilli were thin and increased in number. The differences between the two studies are possibly associated with the differences in how the electron microscopy was performed. Once the cell membrane barrier function of lung tissue is damaged, cell edema is caused. With aggravation of cell injury, various cellular organelles, including the mitochondria, Golgi apparatus and endoplasmic reticula, present corresponding function disorders. In 1992, Marino *et al* (20) observed that when the body temperature was >42°C, intracellular mitochondrial oxidative phosphorylation of skeletal muscle becomes dysfunctional. Another study showed that hyperthermia caused marked reductions in the respiratory control ratio and the oxidative phosphorylation efficacy of rat myocardial cell mitochondria, and the reduction in the Ca^2+^ ATP enzyme activity and Ca^2+^ content of myocardial cell mitochondria caused mitochondrial function disorders (21). This is in line with the observations of the ultrastructure of type II epithelial cells of rats with systemic hyperthermia in the present study. The other mechanism is hyperthermia causes the body to greatly release inflammatory mediators and cells to induce a systemic inflammatory response and immune dysfunction, thus causing SIRS. Zheng *et al* (22) investigated the early inflammatory factor levels of rats with heat stress and identified that the levels of the major proinflammatory cytokines [including tumor necrosis factor (TNF)-α, interleukin (IL)-6, IL-8 and IL-10] in rats with heat stress were increased within 24 h of the establishment of heat stress, and the systemic inflammatory response was evident. These cytokines connect and coordinate with each other to form a complex network system, amplify the inflammatory reaction through a positive feedback mechanism and aggravate lung tissue damage. Among the mechanisms of hyperthermia-induced lung damage, it remains unclear which is the main cause. Naučienė *et al* (23) hypothesized that mitochondrial damage was the main mechanism of action of hyperthermia. The mechanisms of hyperthermia-induced lung damage require investigation in further studies.

UTI is a trypsin inhibitor isolated and purified from human urine. UTI inhibits the activities of a variety of types of proteins, carbohydrates and lipid hydrolases in the body, scavenges oxygen free radicals, relieves local tissue peroxidation, inhibits the synthesis of excess superoxide and myocardial depressant factor, and reduces the excessive release of inflammatory cytokines, resulting in improved microcirculation and immune regulation. UTI has been widely used for treating clinical critical diseases, including severe acute pancreatitis and disseminated intravascular coagulation (11–13). A number of studies have shown that UTI is able to effectively reduce the levels of serum proinflammatory cytokines (TNF-α, IL-1, IL-6 and IL-8) in patients with pyemia and promote the synthesis and secretion of the inhibitory proinflammatory cytokine IL-10 to have a bidirectional regulatory effect on the inflammatory response and thus relieve an excessive inflammatory response (14,24). The results in the present study showed that the W/D values of the HU and HU1 groups, which received early UTI intervention, were significantly lower than those of the H group, and the pathological changes were milder. These results suggest that early UTI application attenuates hyperthermia-induced lung injury and protects the lungs. This is in line with the results of a previous study which demonstrated the protective effect of UTI against acute lung injury in rats caused by endotoxins and LPS (5,15,16). A study (16) hypothesized that the mechanism of action of UTI was associated with the ability of UTI to inhibit TNF-α generation via the p38 mitogen-activated protein kinase signaling pathway at the transcriptional level to affect early acute lung injury, thereby protecting the lung. It is of note that in the present study, the pathological changes of the lung tissue of the rats in the HU2 group with UTI intervention in late stage were evident and similar to those of the H group. It may be speculated that prior to the action of UTI when administered in the late stage, hyperthermia has caused irreversible damage to the cells of the lung tissue. UTI intervention was conducted after 2 h of heat stress in the HU2 group, and the body temperatures of the majority of the rats rose to >42°C by the end of this study. A previous study suggested that when the body temperature is >42°C, intracellular mitochondrial oxidative phosphorylation dysfunction, cell injury and heart failure and heart failure are likely to occur (20). The aforementioned results indicate that UTI should be applied as early as possible for treating and preventing hyperthermia-induced lung damage in the clinic.

During surgery, there are numerous factors that may induce fatal hyperthermia (6–9), including pyemia, thyroid storm, pheochromocytoma and MH. MH (25) refers to the anesthetic-induced abnormally high metabolic status of skeletal muscle and this causes rhabdomyolysis. It is a rare complication of anesthesia with an extremely high mortality rate, and its typical symptoms include masticatory spasm, skeletal myotonia, respiratory acidosis and rapid elevation of body temperature (>38.8°C), as well as plasma creatine kinase elevation and myoglobinuria during anesthesia. Following the exclusion of other potential causes, fatal hyperthermia is diagnosed as MH. Mitochondrial injury plays an important role in the pathological mechanism of MH occurrence (26), and further studies are required to investigate whether UTI has a protective effect on mitochondrial injury and whether early intervention with UTI is effective for treatment of the MH-susceptible population.

To the best of our knowledge, there are no studies on the applications and trials of UTI in rescuing patients with fatal hyperthermia. The results of the present study show that early intervention with UTI relieves the extent of hyperthermia-induced lung tissue cell damage in rats with hyperthermia and has a certain protective effect on the lung. This study provides an experimental basis for the reasonable application of UTI in cases of intraoperative fatal hyperthermia in the clinic.

## Figures and Tables

**Table I:** Comparison of the weight and rectal temperature of the rats at different time points.

		Rectal temperature (°C)					
		
Group (n=8)	Weight (g)	T0	T30	T60	T90	T120	T150
C	185.8±30.5	37.2±0.2	37.1±0.2	37.1±0.2	37.2±0.1	37.1±0.1	37.2±0.4
H	194.5±11.8	37.3±0.2	38.6±0.2	39.0±0.3	39.8±0.3	40.8±0.5	42.3±0.4
HU	194.9±8.3	37.1±0.1	38.5±0.4	38.9±0.4	39.6±0.6	40.6±0.3	42.1±0.4
HU1	192.2±11.7	37.2±0.2	38.7±0.3	39.1±0.3	39.8±0.2	40.6±0.3	42.1±0.4
HU2	190.3±6.8	37.2±0.2	38.6±0.3	38.9±0.3	39.8±0.5	40.7±0.5	42.2±0.5

Values are the mean ± SD. T0, baseline prior to heating; T30, 30 min heat; T60, 60 min heat; T90, 90 min heat; T120, 120 min heat; T150, 150 min heat. No statistical differences in the weights and rectal temperatures were observed among the hyperthermia groups (P>0.05). C, normal control group; H, hyperthermia without medication; HU, hyperthermia and UTI pretreatment; HU1, treated with UTI after 1 h of heating; HU2, treated with UTI after 2 h of heating. UTI, ulinastatin.
